# Artificial Intelligence: Present and Future Potential for Solid Organ Transplantation

**DOI:** 10.3389/ti.2022.10640

**Published:** 2022-07-04

**Authors:** Andrea Peloso, Beat Moeckli, Vaihere Delaune, Graziano Oldani, Axel Andres, Philippe Compagnon

**Affiliations:** ^1^ Department of General Surgery, University of Geneva Hospitals, University of Geneva, Geneva, Switzerland; ^2^ Department of Transplantation, University of Geneva Hospitals, University of Geneva, Geneva, Switzerland

**Keywords:** organ transplantation, machine learning, artificial intelligence, deep learning, result prediction, healthcare 4.0, digital pathology

## Abstract

Artificial intelligence (AI) refers to computer algorithms used to complete tasks that usually require human intelligence. Typical examples include complex decision-making and- image or speech analysis. AI application in healthcare is rapidly evolving and it undoubtedly holds an enormous potential for the field of solid organ transplantation. In this review, we provide an overview of AI-based approaches in solid organ transplantation. Particularly, we identified four key areas of transplantation which could be facilitated by AI: organ allocation and donor-recipient pairing, transplant oncology, real-time immunosuppression regimes, and precision transplant pathology. The potential implementations are vast—from improved allocation algorithms, smart donor-recipient matching and dynamic adaptation of immunosuppression to automated analysis of transplant pathology. We are convinced that we are at the beginning of a new digital era in transplantation, and that AI has the potential to improve graft and patient survival. This manuscript provides a glimpse into how AI innovations could shape an exciting future for the transplantation community.

## Introduction

Artificial intelligence (AI) refers to the use of algorithms (machine learning and deep learning) to perform tasks that are usually associated with human intelligence, “such as the ability to reason, discover meaning, generalize, or learn from past experience to achieve goals without being explicitly programmed for specific action” (1, 2). AI is already changing industry through new forms of interaction between man and machine. Driven by AI, this industrial revolution (known as I4.0) brought intelligent factories where humans and cyber-physical systems interact through deep-learning algorithms. These technologies are increasingly in demand in all industries which seek to ensure manufacturing competitiveness.

Powered by increasing availability of healthcare data and rapid development of analytical techniques, AI is also growing exponentially in all areas of medicine, including solid organ transplantation. The pre- and post-transplantation patient care requires complex decision-making. In this context, AI can drive a real paradigm shift as it enables analyzing and synthesizing of huge amounts of data, and transforming them into clinical recommendations. AI-based classifiers have been principally explored for the optimization of four key areas: organ allocation and donor-recipient pairing, transplant oncology, real-time immunosuppression regimes, and precision transplant pathology. The aim of AI is to identify hidden trends and complex relationships within large datasets to obtain logical results while optimizing resources. AI is still in its infancy and, so far, we lack validated algorithms that could accurately drive organ selection, predict potential rejections or attenuate postoperative complications. Nevertheless, in the last few decades, AI applications have already contributed to lower incidence of rejection, and fine-tuning of the transplantation and organ preservation processes. In this review, we discuss emerging AI, machine learning and deep learning strategies applied to solid organ transplantation and their potential future applications (Table 1).

**TABLE 1:** AI Glossary table.

Term	Definition
Computer Algorithms	Computer algorithms are automated instructions
Machine Learning (ML)	Machine learning is a subfield of artificial intelligence intended as a sets of automated computer algorithms
Deep-Learning (DL)	Deep learning is a type of ML that imitates the way humans gain certain types of knowledge including statistics and predictive modeling
Neural Networks (NN)	Neural networks reflect the behavior of the human brain, allowing computer algorithms to recognize patterns and solve common problems in the fields of AI, ML and DL.
Cyber Physical System	Cyber Physical System is referred to computer-human networks, controlling physical processes, where physical processes affect computations and vice versa
Internet of Things	The Internet of Things represents a system of interralated computing devices, capable of operating without human-to-human or human-to-computer interaction

## AI in Organ Allocation and Donor-Recipient Matching Modeling

From an exclusively mathematical point of view, transplantation can be reduced to a list of problems in which the characteristics of the donor must be combined with the variables of the recipient in order to achieve one of the following three outcomes (2): the survival of the graft and the recipient, the loss of the graft or the loss of the graft and the recipient.

The allocation systems used by Eurotransplant in Europe, and the United Network for Organ Sharing (UNOS) in the US, are intended as objective and transparent procedures to make the best possible match (3, 4). The allocation systems, which are one of the cornerstones of transplantation, are based on two major principles: expected outcome and emergency. Additionally, the allocation (and donor-recipient matching) process depends on the timeframe during which the organ remains viable once harvested, which ranges from a few to 36 h, depending on the organ (5). Organ-matching characteristics may differ between organs, but they are crucial for the selection of the best possible allocation and donor-recipient matching. The Child-Pugh classification, the Model of End Stage Liver Disease (MELD), the Kidney Allocation System (KAS) and the Lung Allocation System (LAS) are the most important algorithms currently used (6). Whilst well integrated into clinical practice, these systems cannot prioritize recipients in real time and need constant modifications (7) and addition of exceptions. AI could significantly strengthen the decision-making, by automatically harmonizing principles of optimal use (utility) and equal access (equity) in a context of organ shortage and an ever-growing waiting list. In 2019, Bertsimas and co-workers proposed a machine learning-based model for alternative liver allocation (8). This model, named Optimal Prediction of Mortality (OPOM), predicts the probability of a patient’s 3-month mortality or waitlist removal given their characteristics. Using the Standard Transplant Analysis and Research dataset (1618966 observations), OPOM provided more accurate and objective predictions than MELD. Additionally, the OPOM simulation reduced mortality on average by 417.96 deaths for 6139 liver transplantations by assigning different priority to liver transplant candidates. External validation still needs to be performed.

**FIGURE 1:**
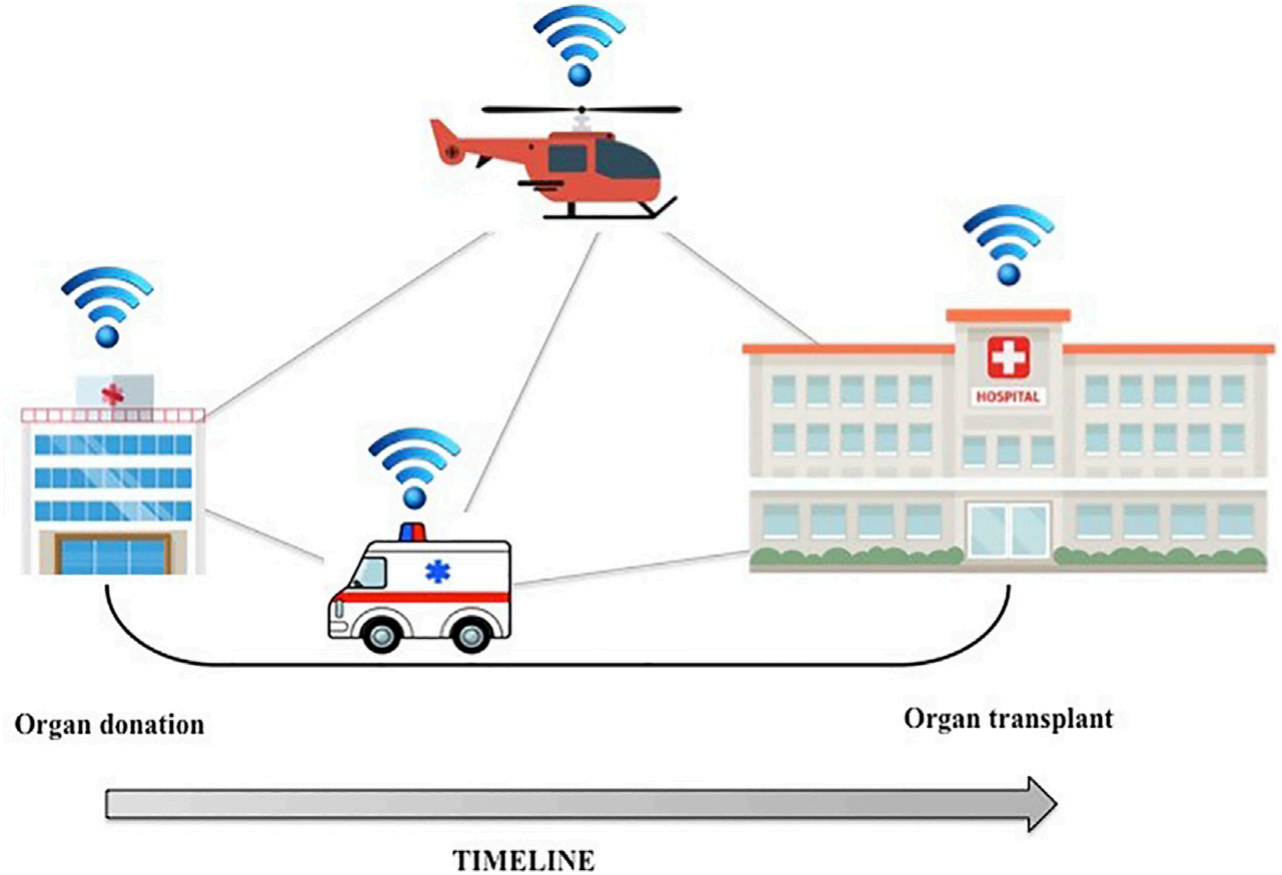
GPS tracking technology could be applied to organ transport, thus minimizing downtime and optimizing the workflow.

Organ allocation could also benefit from the Internet of Things (IoT). IoT refers to a network of interconnected smart devices such as smartphones, tablets, and laptops, but also wearables, cars, and data transmission devices (9). An IoT ecosystem of web-enabled connected devices using sensors, processors and communication hardware can be used to store, transmit and react appropriately to data from the surroundings. During the organ procurement and transplantation process, the distance between the donor and the recipient is a key factor influencing the time needed for organ transfer. Even if routinely preserved in ice-cold preservation fluids, organs are sensitive to cold ischemia time. IoT could be useful for real-time tracking of organs: during transport, the organ packaging can be equipped with a global positioning system (GPS) that can continuously track the organ’s location and record shocks caused by rapid acceleration/deceleration or barometric pressure incidents (10). These data can be used to accurately approximate time of organ arrival in the recipient’s transplant center, minimize downtime and optimize the workflow (Figure 1).

Organ allocation is strictly connected to donor-recipient matching. Although thoroughly analyzed and refined, the traditional donor-recipient matching models still leave room for improvement and could potentially benefit from AI. In 2013, Cruz-Ramirez et al. reported the use of AI artificial neural networks (AI-ANNs) to improve donor-recipient pairing. AI-ANNs analyzed data on 1,003 liver transplants including donor/recipient matching, graft retrieval and pre-transplant analysis (11). The following year, a large Spanish multicenter study (Model for Allocation of Donor and Recipient in España [MADR-E]) documented the impressive advantages of using AI-ANNs rather than standard algorithms (12). In their work, Briceño et al. designed a 3-month graft mortality prediction model based on 64 donor and recipient characteristics and performed a binary analysis (graft survival/loss) for donor-recipient matching via AI-ANNs. AI-ANNs’ new algorithms predicted graft survival (AUC, 0.81) and graft loss (AUC, 0.82) better than the isolated donor/recipient scores. Similarly, Rana and coworkers used the Organ Procurement and Transplantation Network (OPTN)/Scientific Registry of Transplant Recipients (SRTR) data to develop the Survival Outcome Following Liver Transplant (SOFT) score that integrates recipient and donor characteristics to predict liver transplant 3-month prognosis (13). The SOFT score demonstrated a predictive accuracy similar to those of other models (14, 15) with a C-statistic of 0.70.

The reports on impact of diabetes on the outcome of liver transplantation have been contradictory. Recently, Yasodhara et al. demonstrated the value of AI for successful liver donor-recipient matching, by including the metabolic status of the recipient (16). Based on the SRTR registry, the authors used machine learning to establish survival predictors in liver transplant recipients with preexisting and/or post-transplant diabetes. They tested survival models to predict general and cardiovascular mortality and evaluated the effects of preexisting and post-transplant diabetes on mortality. The model performance achieved C-statistics between 0.58 and 0.66. Additionally, the model was externally validated on a cohort of patients (University Health Network dataset from Toronto, Canada). While the study had some limitations (retrospective design, missing data on patients’ comorbidities, unclear information regarding immunosuppression, unusually few patients with steatohepatitis), it is nevertheless one of the largest studies to address risk factors in liver transplant patients with diabetes. AI and machine learning enabled the authors to analyze the huge and heterogeneous dataset and conclude that diabetes is a superior predictor of outcome than obesity, which resulted in changes in practice in donor-recipient matching.

AI-ANNs algorithms have been also applied to donor-recipient matching for kidney transplants. In 2019, Bae et al. proposed an online tool (https://www.transplantmodels.com/kdpi-epts/) (17) to maximize benefits form marginal kidney donors. The authors estimated the 5-year patient survival using a random survival forest (RSF), with the combination of expected post-transplant survival (EPTS) score (variables: age, diabetes, time on dialysis and previous solid organ transplant) and Kidney Donor Profile Index (KDPI) (variables: age, race, height, weight, hypertension, diabetes, serum creatinine, hepatitis-C seropositivity and cause of death). The result of the evaluation yielded a C-statistic of 0.637 for the RSF algorithm, which is slightly higher than the Kidney Donor Risk Index (KDRI) model’s 0.6. This prediction model could support personalized decision-making on kidney offers in clinical practice.

## AI in Transplant Oncology

Transplant oncology is defined as a combination of various fields of transplant medicine and oncology, aiming to extend the treatment limits of hepatobiliary cancer including hepatocellular carcinoma (HCC), cholangiocarcinoma or colorectal liver metastases (18, 19).

In the past this discipline relied on simple variables such as the number of tumor lesions and their size. In recent years, transplant oncology was refined and a multitude of new variables identified as central, making AI a potentially important tool. Identification of key clinical and pathological variables is a crucial step in the use of AI for the prediction of tumor recurrence and graft survival after transplantation (20). AI has been used by different groups to predict oncological outcomes in patients undergoing liver transplantation for HCC. Halazun KJ et al. developed a model (MORAL - Model of Recurrence after Liver Transplant) which identified predicting factors of tumor recurrence pre- and post- liver transplantation (21). Specifically, neutrophil-lymphocyte ratio ≤5, alpha-fetoprotein (AFP) > 200 ng/ml and tumor size >3 cm have been classified as pre-transplantation predictive factors of decreased recurrence-free survival. Likewise, HCC grade 4, tumor size >3 cm, > 3 tumor lesions, and vascular invasion have been identified as post-transplantation negative predictive factors. Both scored (pre- and post-transplant) demonstrated predictive superiority (C-statistic of 0.82 and 0.86, respectively) when compared to Milan criteria for forecasting tumor recurrence (C-statistic of 0.63). When combined, the two scores achieved a C-statistic of 0.91.

The Metroticket 2.0 score proposed by Mazzaferro and coworkers (22) predicts survival after liver transplantation for HCC through competing-risk analysis. The authors enrolled 1018 patients from an internal cohort in Italy, while the score was validated by an external Chinese cohort of 341 patients. Preoperative characteristics such as AFP level, tumor volume and number of tumors were included. The validation set showed an accuracy of 0.721 (95% CI, 0.648%–0.793%) in predicting 5-year survival after liver transplant. This model was compared to Milan, Up-to-7 and UCSF criteria, demonstrating a superior predictive ability.

Recently, the group lead by Prof. Sapisochin described the use of AI for predicting the post-transplant recurrence of HCC based on preoperative patient and tumor characteristics (23). To do this, the group included HCC patients listed for liver transplantation between 2000 and 2016 (*n* = 739). This AI-based HCC-recurrence calculator (CoxNet-based) was then compared to alternative available recurrence risk scores (AFP, MORAL and HALT-HCC scores). The CoxNet-based algorithm outperformed AFP by 0.118, MORAL by 0.130 and HALT-HCC by 0.102. These findings confirm, pending an external validation, that an AI-based calculator can generate a comprehensive prediction of post-transplant HCC recurrence with higher accuracy than alternative scores.

## AI and Real-Time Adaptation of Immunosuppressive Therapy

The discovery of cyclosporine was a cornerstone of modern transplantation (24) and constant refinement of immunosuppressive regimens drastically improved outcomes for transplant patients (25). However, immunosuppressive regimens are burdened with adverse effects ranging from nephrotoxicity to malignancies, and significantly reduce the quality of life and life expectancy of transplant patients (26, 27). Furthermore, response to immunosuppressive therapy is highly individual. While some patients do not require any immunosuppression at all, others reject their organs on maximum immunosuppression (28–30). The individual optimization of immunosuppression is therefore of the utmost importance.

Many factors come into play when choosing the optimal immunosuppression regimen, and the decision-making is complex. One relatively simple example of machine learning use is to predict the stable dose of tacrolimus in kidney transplant patients. Three studies compared the logistic regression approach to machine learning algorithms (31–33). All studies showed a superior predictive ability of machine learning tools over the linear regression models, albeit with a relatively small difference. Using combination of genomic data and clinical factors was shown more important than the choice of algorithm. The improved prediction performance highlights the importance of integrating data from different sources (31).

Taking a more general approach, Nitski et al. analyzed large retrospective datasets with machine learning algorithms to predict mortality in liver transplant patients (34). The models were longitudinally updated with patients’ data at every follow-up. Interestingly, the model provided meaningful predictions based on readily available data such as graft age, blood values, donor age, and postoperative complications, making a potential clinical implementation relatively straightforward. This dynamic model could be a valuable tool for clinicians to personalize immunosuppressive therapy based on the most likely complication, and therefore reduce graft-related mortality (35). Biomarkers surveillance plays an important role in predicting transplant rejection in patients on immunosuppression.

Suthanthiran *et al.* used the transcriptomes of urinary cells from 220 patients to predict acute rejection based on kidney biopsies (36). The authors obtained an AUC of 0.85 with a three-gene expression signature for the discrimination between acute rejection and no rejection in their own cohort, and an AUC of 0.74 upon external validation. However, the authors used a predefined gene set, while a genome-wide association study would have likely revealed better gene candidates (37). Deep learning tools could have been helpful in this big-data context to not only find these candidates but also to further improve the already working prediction model (38).

AI can integrate high-complexity information from many sources into the decision-making tree used in individualized immunosuppression. A wealth of information about donors and recipients is still underutilized. Data from pre-transplantation histology, recipient’s genome, gene expression analysis, blood and urine analysis, and clinical observation can all deliver important clues on the state of a transplanted organ (39–41). AI can help us tap into this potential to fine-tune immunosuppression, and optimize graft and patient survival.

## AI in Transplant Pathology

AI has proven highly efficient in image processing. An image contains a high density of structured and unstructured information that is often inaccessible to the untrained eye (42). A pathologist has the experience and the training to recognize subtle patterns and interpret them in the context of a particular patient and their disease. Unfortunately, trained pathologists are in short supply. This is where AI steps in to extract, process, analyze and even learn from the wealth of information contained in pathological slides (1), that can guide therapy or improve diagnostic accuracy. More than 2 decades ago, Furness et al. developed a machine learning algorithm that diagnosed the acute kidney allograft rejection more accurately than expert pathologists (43). However, the algorithm was not fully automated—it relied on manual extraction of pathological features from histological slides. This method of data collection illustrates why AI did not find a more widespread application in transplant pathology sooner: collecting raw data is a prerequisite for downstream analysis. Commercial digital pathology slide scanners for high-throughput imaging have only recently become available (44, 45). Advances in computer performance, data storage and network speed enable increasingly efficient analysis. The I4.0 now provides us with the tools to fully exploit the potential of AI in transplant pathology. In a recent study, Hermsen et al., successfully implemented a deep learning algorithm to divide kidney biopsies from different centers into their anatomical components (46). The authors developed a convolutional neural network that classified each anatomical component. While the algorithm performed well in identifying healthy glomeruli, it struggled to identify more challenging structures such as sclerotic glomeruli or atrophic tubuli. Nevertheless, this study provides important groundwork and paves the way for further image analysis of kidney transplant biopsies. Most importantly, the authors proved that the same algorithm worked on histological samples from different centers, thereby addressing the issue of reproducibility.

In liver transplantation, quick and reliable assessment of liver steatosis during procurement still presents a challenge. Recently, several groups have developed deep learning algorithms to assess the degree of steatosis in liver biopsies (47–49). Perez-Sanz et al. developed a quick and easy workflow to quantify steatosis content in Sudan-stained frozen sections of procurement biopsies through machine learning. Their algorithm, available as an open-source interactive web platform (50), proved highly accurate in comparison with the assessment of an expert pathologist. This tool could be extremely valuable for the decision-making in remote procurement locations, where an expert pathologist is not readily available.

Automated image analysis, feature recognition, data extraction and deep learning models are everyday reality for the tech giants but have only partially reached precision pathology (51–53). Radiology is one step ahead and shows what is possible with the emerging field of radiomics—the extraction of data from radiograms to diagnose cancer, predict outcomes or guide therapy (54–56). Transplant pathology needs to follow this example with a concerted, multidisciplinary effort of pathologists, computational biologists and healthcare administrators. Challenges that lie ahead are the implementation of digital workflows to routinely scan histological slides, and collaboration between centers to establish image databases and bring the existing AI tools to transplant pathology (57).

## Conclusion: AI Current Pitfalls and Future Promises

The true potential of AI in healthcare has yet to be fully exploited and its application in solid organ transplantation is mostly under development. Some important limitations exist (58). Several algorithms have been developed in a single institution and still need an external validation to prove their robustness. Secondly, in some cases, the use of AI cannot provide significant improvements over current models (58–60). Moreover, the creation of a more comprehensive AI-based decision model (which includes characteristic of all organs as well patient-specific alternative therapeutic strategies) should be targeted. On the one hand, this could bring new insights to potentially enlarge the pool of transplantable organs and, on the other, improve patient outcomes. Implementing AI into daily clinical practice is an ongoing challenge and the best strategy forward is unclear. While most physicians are unconvinced that can AI play a weighty role in medicine, it is naive to think that this technology will not develop further. Moreover, while this manuscript focuses on the use of AI in transplantation, many other domains could benefit from it. Precision medicine (genetic-based solutions, drug discovery and development) (61), AI-assisted computer vision (62), augmented and virtual reality (63) and the AI-assisted integration and collection of patients’ records (64, 65) are just few examples of how AI can be applied to medicine. AI is on a trajectory of exponential growth, and has the potential to improve how we experience our lives and to extend life itself.
